# Supplementary material for: Feed Supplemented with Aronia melanocarpa (AM) Relieves the Oxidative Stress Caused by Ovulation in Peak Laying Hens and Increases the Content of Yolk Precursors
Source: Animals (Basel). 2022 Dec 17;12(24):3574. doi: 10.3390/ani12243574 (PMC9774901; doi:10.3390/ani12243574)
Supplement: Supplementary file 1 [file animals-12-03574-s001.zip › animals-2061885-supplementary.pdf]

Supplementary Table:

Table S1. Composition and nutrient levels of diets.

Item, %	control	VC	1%AM	4%AM	7%AM
Corn	63.150	63.150	61.930	58.190	54.490
Soybean meal	25.700	25.700	25.860	26.330	26.800
Stone powder	8.930	8.930	8.930	8.930	8.930
Steamed bone meal	0.700	0.700	0.700	0.700	0.700
Soybean oil	0.750	0.750	0.810	1.080	1.310
Salt	0.300	0.300	0.300	0.300	0.300
Choline chloride	0.100	0.100	0.100	0.100	0.100
Vitamin–mineral premix[a]	1.000	1.000	1.000	1.000	1.000
Methionine	0.120	0.120	0.120	0.120	0.120
Lysine	0.100	0.100	0.100	0.100	0.100
Vitamin C		0.05			
Aronia melanocarpa			1	4	7
Total	100.85	100.90	100.85	100.85	100.85
Nutrient level, %					
Metabolizable energy (MC/Kg)	11.30	11.30	11.30	11.30	11.30
Crude protein	17.40	17.40	17.40	17.40	17.40
Ca	3.36	3.36	3.36	3.36	3.36
Lys	0.95	0.95	0.95	0.95	0.95
Met	0.39	0.39	0.39	0.39	0.39
Met+Cys	0.30	0.30	0.30	0.30	0.30
Crude fiber	2.55	2.55	2.58	2.68	2.78
Available phosphorus	0.10	0.10	0.10	0.10	0.10
NaCl	0.29	0.29	0.29	0.29	0.29

Trace elements and vitamin are provided by the Mingqian Green Layer Breeding Professional Cooperative in Liaoyuan City, Jilin Province.

Note: The nutrient content in feed formulation is theoretical calculation values.

[a] Vitamin-mineral premix provide (per kg diet): 25,300 IU vitamin A, 7,000 IU vitamin D 3, 270 IU vitamin E, 45 mg vitamin K3, 32 mg vitamin B1, 110 mg vitamin B2, 30 mg vitamin B6, 0.6 mg vitamin B12, 40 mg niacin, 13 mg pantothenic acid, 13 mg folic acid, 0.8 mg biotin, 100 mg choline chloride, 60 mg Fe,1.4 mg Cu, 60 mg Mn,13 mg Zn, 0.012 mg Se, 0.098 mg I.

Table S2. Primer sequences of genes selected for analysis by RT-qPCR

Target gene	Accession number	Primer sequence (5′–3′)	Product size
β-actin	NM_205518.1	Forward: AACCGGACTGTTACCAACACC	156bp
		Reverse: AGACTGCTGCTGACACCTTCAC	
Keap1	XM_025145847.1	Forward: AGCAGCGTGAGAGGTGAGTATG	113bp
		Reverse: GCGTACAGCAGTCGGTTCAG	
Nrf2	NM_205117.1	Forward: GGGACGGTGACACAGGAACAAC	97bp
		Reverse: GCTCTCCACAGCGGGAAATCAG	
HO-1	NM_205344.1	Forward: GCTGGGAAGGAGAGTGAGAGGAC	107bp
		Reverse: GCGACTGTGGTGGCGATGAAG	
SOD1	NM_205064.1	Forward: TCTTACCGGACCACACTGCATC	115bp
		Reverse: ACGAGGTCCAGCATTTCCAGTTA	
apoB	NM_001044633.1	Forward: ACACTTCGGGCTATTGGA	129bp
		Reverse: TGCCTGTATGGCTGCTTT	
apoVLDLII	NM_205483.2	Forward: AGCAGGACAGCAGGTCTCTTGG	114bp
		Reverse: TCAGGGACAGTGGTGCTAAGGAG	
VTGII	NM_001031276.1	Forward: TTCGAAGCTGATGAACACACAC	192bp
		Reverse: GATTGCTTCATCTGCCAGGTC	
VLDLR	NM_205229.1	Forward: GTGCGGCTCAGGTGAATGTATCC	97bp
		Reverse: GGTCCGAGAAGGGCAGTTGATTTC	
ER-a	NM_205183.2	Forward: TAGTTCCGCTCTACGACCTCTT	106bp
		Reverse: AGTTGGTTTCGGTTCTCCTCTT	
